# Spent Yeast Waste Streams as a Sustainable Source of Bioactive Peptides for Skin Applications

**DOI:** 10.3390/ijms24032253

**Published:** 2023-01-23

**Authors:** Eduardo M. Costa, Ana Sofia Oliveira, Sara Silva, Alessandra B. Ribeiro, Carla F. Pereira, Carlos Ferreira, Francisca Casanova, Joana O. Pereira, Ricardo Freixo, Manuela E. Pintado, Ana Paula Carvalho, Óscar L. Ramos

**Affiliations:** 1 Escola Superior de Biotecnologia, CBQF—Centro de Biotecnologia e Química Fina—Laboratório Associado, Universidade Católica Portuguesa, Rua Diogo Botelho 1327, 4169-005 Porto, Portugal; 2 Amyris Bio Products Portugal Unipessoal Lda, 4169-005 Porto, Portugal

**Keywords:** spent yeast, skin application, collagen IαI, hyaluronic acid, waste stream valorization

## Abstract

Spent yeast waste streams are a byproduct obtained from fermentation process and have been shown to be a rich secondary source of bioactive compounds such as phenolic compounds and peptides. The latter are of particular interest for skin care and cosmetics as they have been shown to be safe and hypoallergenic while simultaneously being able to exert various effects upon the epidermis modulating immune response and targeting skin metabolites, such as collagen production. As the potential of spent yeast’s peptides has been mainly explored for food-related applications, this work sought to understand if peptide fractions previously extracted from fermentation engineered spent yeast (*Saccharomyces cerevisiae*) waste streams possess biological potential for skin-related applications. To that end, cytotoxic effects on HaCat and HDFa cells and whether they were capable of exerting a positive effect upon the production of skin metabolites relevant for skin health, such as collagen, hyaluronic acid, fibronectin and elastin, were evaluated. The results showed that the peptide fractions assayed were not cytotoxic up to the highest concentration tested (500 µg/mL) for both cell lines tested. Furthermore, all peptide fractions showed a capacity to modulate the various target metabolites production with an overall positive effect being observed for the four fractions over the six selected targets (pro-collagen IαI, hyaluronic acid, fibronectin, cytokeratin-14, elastin, and aquaporin-9). Concerning the evaluated fractions, the overall best performance (Gpep > 1 kDa) was of an average promotion of 41.25% over the six metabolites and two cell lines assessed at a concentration of 100 µg/mL. These results showed that the peptide fractions assayed in this work have potential for future applications in skin-related products at relatively low concentrations, thus providing an alternative solution for one of the fermentation industry’s waste streams and creating a novel and highly valuable bioactive ingredient with encompassing activity to be applied in future skin care formulations.

## 1. Introduction

Peptides have long been favored for use by the cosmetic industry with the reports of the development of synthetic peptides dating back to the early 1980s with the synthesis and application of copper glycine–histidine–lysine peptide being described [1,2]. However, during the last 20 years peptide application in cosmetics has been residual, and the first real application of peptides as an active cosmetic agent appeared in the early 2000s with the synthesis and launch of the palmitoyl pentapeptide-4 peptide as an anti-wrinkle agent [2].

Currently, peptides are considered to be one of the go-to solutions for the cosmetic industry as they are biocompatible, bioactive, and capable of interacting with various aspects of the cellular machinery modulating cellular response. Being one of the go-to solutions, a set of desirable properties has been drawn for peptides based on a perceived skin permeation capacity. This has led to cosmetic industries searching and synthesizing peptides with molecular weights (MWs) below 500 Da, octanol/water partition coefficients between 1 and 3, and water solubility above 1 mg/mL [2,3]. However, this set of characteristics and the importance of the MW has been recently put in question as some works have shown that skin permeability is, in reality, a complex system where the hydrophobic or hydrophilic nature of the solute plays a key role. In fact, the equilibrium between various different stratum corneum permeation routes (volume diffusion through lipid bilayers, lateral diffusion along lipid bilayers, diffusion through pores, and diffusion through shunts) has been shown to be the real factor differentiating compound permeation, thus allowing for the usage of different molecules diverging from the previously defined template [4,5]. Additionally, another factor that must be considered is peptides’ susceptibility to skin proteolytic enzymes as they use peptides as substrates for their activity [6].

In recent years, with the drive towards sustainability and eco-friendliness, the need for new and natural sources of bioactive compounds has driven research into exploring and valorizing alternative sources or alternative molecules way streams. This has resulted in byproducts rising to prominence as alternative sources of bioactive-rich compounds that can be used by the various industries. When considering peptides and possible alternative sources, fermentation industry engineered spent yeast waste streams (SYWS) have risen to prominence in later years as it is considered as being Generally Recognized As Safe (GRAS), as it is mainly constituted by proteins with biological value (45 to 60% of its dry weight), and its extract has been sold for more than a century [7]. Furthermore, yeast-derived peptides have long been proven to possess several biological properties, such as antihypertensive, antioxidant, and anti-stress effects; immunomodulation; and in vivo promotion of bone growth, which makes them interesting for the cosmetic industry [7,8,9,10,11,12].

With this in mind, we hypothesized that peptide fractions previously extracted from engineered spent yeast waste streams possess cosmetic potential and are able to modulate the production of cosmetic relevant metabolites in vitro. To that end, spent yeast waste streams of β-glucan and mannan extraction were purified through a 1 kDa cut-off membrane ultrafiltration, as previously described by Oliveira, Pereira [7], and four peptide fractions were produced. These fractions were then characterized for their antioxidant capacity and cytotoxic profile towards human skin keratinocytes and fibroblasts, followed by their capacity to modulate the production of various cosmetic relevant metabolites among which are pro-collagen IαI, hyaluronic acid, elastin, and fibronectin. 

## 2. Results

### 2.1. Antioxidant Activity Results

**Figure 1:**
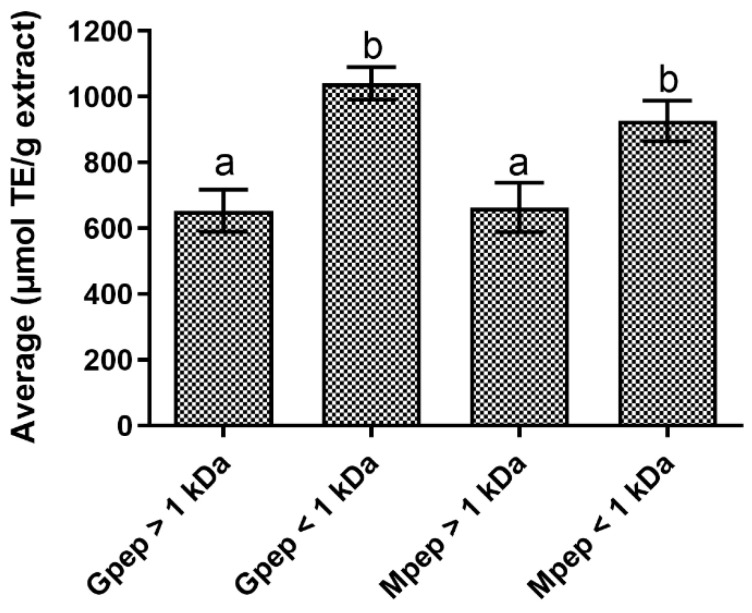
Peptide fractions antioxidant activity according to the ORAC assay. Different letters represent the statistically significant (*p* < 0.05) differences found between the tested samples at the tested concentration.

The results obtained for the studied peptide fractions regarding antioxidant potential showed (Figure 1) that a higher antioxidant potential was obtained for the two fractions with lower MW, with both presenting statistically significant (*p* < 0.05) differences compared to those above 1 kDa. These results indicate that the peptides fractions’ antioxidant capacity may be directly linked with their MW and that the production method employed was not a factor at play and did not influence the overall antioxidant capacity of the samples. 

### 2.2. Cytototixicity Results

**Figure 2:**
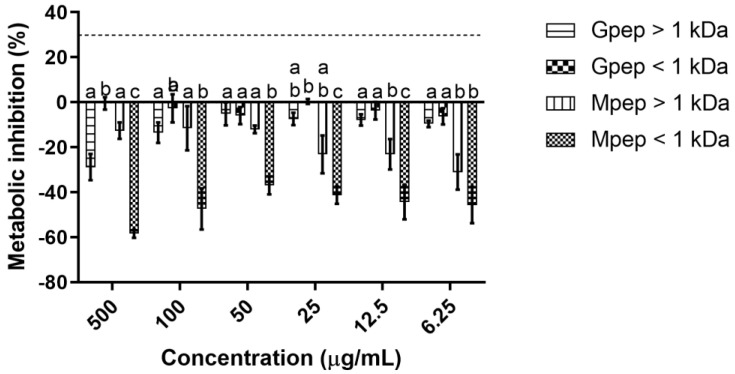
Peptide fractions impact upon HaCat cells metabolic activity. Different letters represent the statistically significant (*p* < 0.05) differences found between the four samples tested at each concentration assayed. The dotted line represents the 30% cytotoxicity limit as defined by the ISO 10993-5:2.

**Figure 3:**
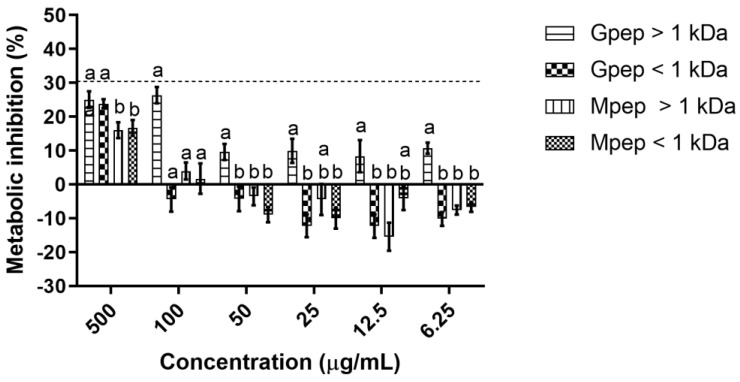
Peptide fractions’ impact upon HDFa cells metabolic activity. Different letters represent the statistically significant (*p* < 0.05) differences found between the four samples tested at each concentration assayed. The dotted line represents the 30% cytotoxicity limit as defined by the ISO 10993-5:2.

The results obtained regarding the cytotoxicity of the assayed peptide fractions against the selected skin cell lines can be seen in Figure 2 and Figure 3. For HaCat (Figure 2), the assayed peptide fractions had no significant impact upon these cells metabolic activity, as at all tested concentrations no reductions in metabolism were observed. In fact, all fractions led to promotions in HaCat metabolism, with the only exception being the Gpep < 1 kDa fraction, which had a non-statistically significant (*p* > 0.05) impact in the cellular metabolism. On the other hand, the Mpep < 1 kDa fraction had a strong impact upon this cell line metabolism, as all tested concentrations of this fraction registered significantly (*p* < 0.05) higher metabolism promotion values than the other fractions. 

When considering the impact upon human fibroblasts (Figure 3), the impact presented is quite different. The first major takeaway is that contrary to what was observed for HaCat, the fractions assayed clearly impacted HDFa metabolism with the highest concentrations tested (500 µg/mL) leading to statistically significant (*p* < 0.05) reductions in the assayed cells metabolism. For the remainder of the concentrations tested, significant (*p* < 0.05) metabolic inhibitions were only observed for the Gpep > 1 kDa, with all other fractions presenting either no alterations or small promotions in the cell’s metabolism. More importantly, despite the inhibitions observed, a cytotoxic effect was not observed as the threshold of 30 defined by the ISO 10993-5:2009 standard [13] was never reached.

### 2.3. Pro-Collagen I α I Biosynthesis Results

**Figure 4:**
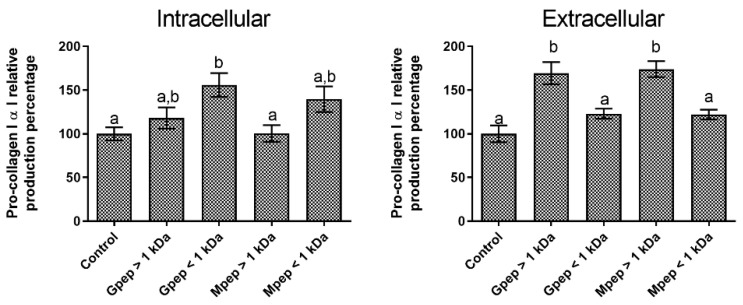
Pro-collagen I α I relative percentage of production in the presence of different peptide fractions relative to the basal level (control) in HDFa cells. Different letters represent the statistically significant differences (*p* < 0.05) found between the samples tested in each setting.

When considering the impact of the peptide fractions upon pro-collagen I α I production, the obtained results (Figure 4) showed that the peptide fractions could modulate this molecule biosynthesis in the two assayed settings. In the intracellular setting (Figure 4A), the best results were obtained for the Gpep < 1 kDa fraction, as it was the only one for which statistically significant (*p* < 0.05) differences relative to the control were obtained. For the other tested fractions, no statistically significant differences (*p* > 0.05) relative to the control were found, and it is particularly interesting to note that for fractions with MWs above 1 kDa, promotions were small or non-existent. On the other hand, when considering the extracellular results obtained (Figure 4B) the data showed a complete opposite pattern of response, as in this setting the fractions with MWs above 1 kDa presented significantly higher (*p* < 0.05) levels of pro-collagen I α I than the basal control. While not directly comparable, it is still worth noticing the higher levels of relative production registered for the extracellular setting versus the intracellular.

### 2.4. Hyaluronic Acid Biosynthesis Results

**Figure 5:**
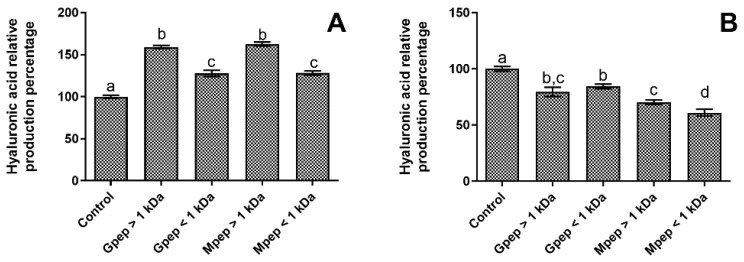
Hyaluronic acid relative percentage of production in the presence of different peptide fractions relative to the basal level (control). (**A**) HDFa; (**B**) HaCat. Different letters represent the statistically significant differences (*p* < 0.05) found between the samples tested in each setting.

The data obtained regarding the studied peptide fractions capacity to modulate hyaluronic acid metabolism can be seen in Figure 5. As can be observed, the results obtained for HDFa cells (Figure 5A) showed that all fractions were capable of stimulating the target metabolite production with statistically significant (*p* < 0.05) increases being registered. Interestingly, results can be grouped by MW, with no statistically significant (*p* > 0.05) differences being observed between fractions with the same MW, but with statistically significance (*p* < 0.05) being found between MWs with fractions higher than 1 kDa presenting significantly higher relative production values than those below 1 kDa.

On the other hand, when considering the hyaluronic acid production modulation in HaCat cells (Figure 5B) peptide fractions were deleterious for the overall levels of this metabolite production, with all fractions presenting statistically significant (*p* < 0.05) lower production percentages than the basal control. In fact, the Mpep < 1 kDa fraction originated reductions of almost 50% relative to the basal control and when analyzing the data, no clear pattern of response could be observed. 

### 2.5. Fibronectin Biosynthesis Results

**Figure 6:**
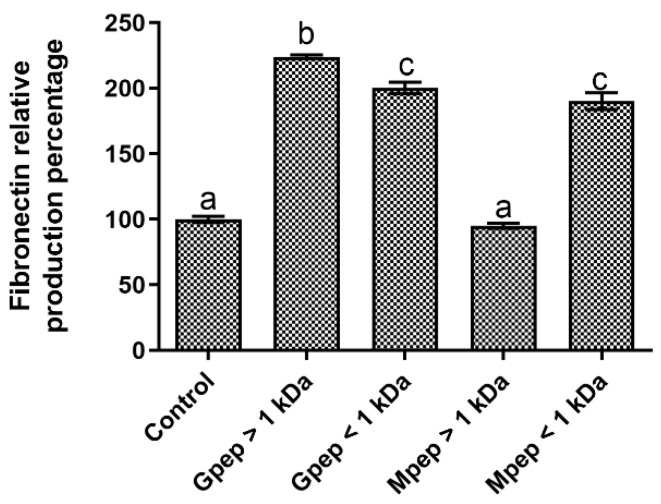
Fibronectin relative percentage of production in the presence of different peptide fractions relative to the basal level (control). Different letters represent the statistically significant differences (*p* < 0.05) found between the samples tested.

For fibronectin production modulation, the obtained data (Figure 6) did not allow to infer any particular pattern associated with the characteristics of the peptide fractions assayed. In fact, with the exception of the Mpep > 1 kDa fraction, all other fractions led to significant (*p* < 0.05) promotions of fibronectin production relative to the basal control. Among the metabolism-promoting fractions, higher values were obtained for the Gpep > 1 kDa, as it presented statistically significantly (*p* < 0.05) higher relative promotion values than the other two; however, no differences (*p* > 0.05) were found between them. Furthermore, it must be noted that the worst results obtained were not deleterious to the overall production of the target metabolite, as the Mpep > 1 kDa fraction was not statistically different (*p* > 0.05) from the control.

### 2.6. Cytokeratin-14 Biosynthesis Results

**Figure 7:**
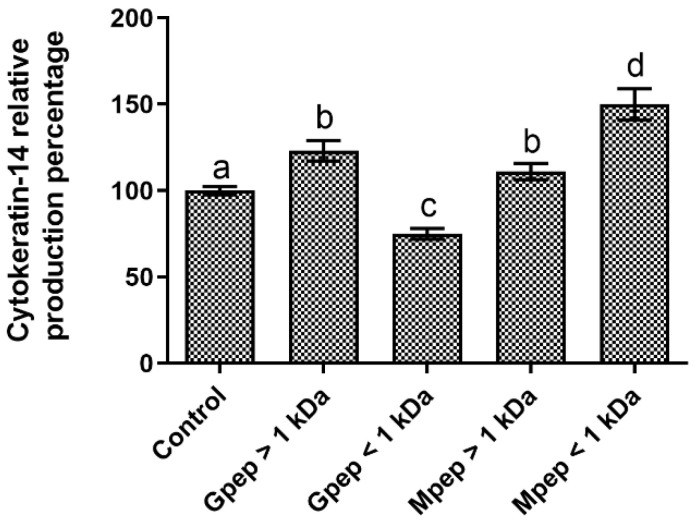
Cytokeratin-14 relative percentage of production in the presence of different peptide fractions relative to the basal level (control) in HaCat cells. Different letters represent the statistically significant differences (*p* < 0.05) found between the samples tested.

When considering the impact of the studied peptide fractions upon the cytokeratin-14 production in HaCat cells, the data obtained (Figure 7) showed that statistically significant (*p* < 0.05) differences were found between all fractions and the basal control. Of the studied fractions, only Gpep < 1 kDa presented reductions in cytokeratin-14 production relative to the control, with the remaining ones presenting different levels of production stimulation. The best performance was observed for Mpep < 1 kDa; however, no clear patterns of response were found when considering the MWs and production methods assessed in this work.

### 2.7. Elastin Biosynthesis Results

**Figure 8:**
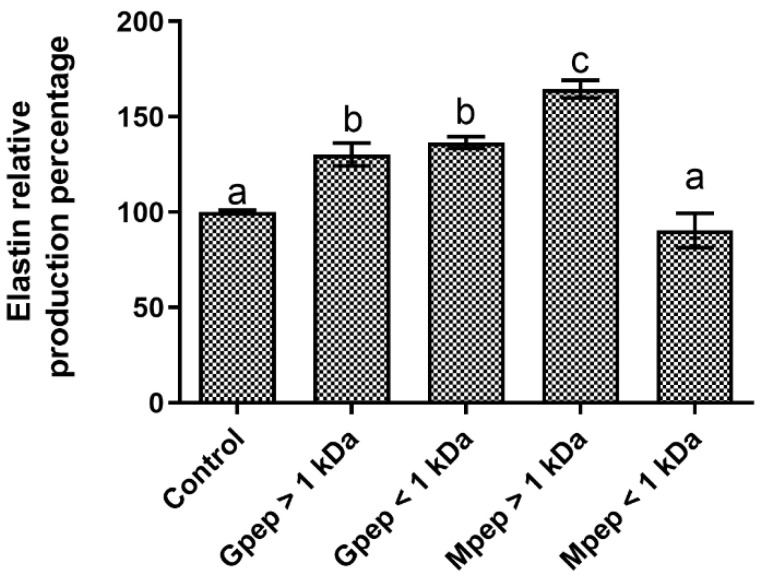
Elastin relative percentage of production in the presence of different peptide fractions relative to the basal level (control) in HDFa cells. Different letters represent the statistically significant differences (*p* < 0.05) found between the samples tested.

The results obtained in the elastin production modulation assays (Figure 8) showed that among the four fractions assayed, three were capable of positively modulating the target metabolite production, with statistically significant (*p* < 0.05) promotions relative to the basal control being registered in these conditions. The only condition for which no promotion was observed (Mpep < 1 kDa) presented a small, not statistically significant (*p* > 0.05) reduction relative to the basal control. Once again, analysis of the results did not show any clear pattern of response associated that could be linked with the characteristics of the studied fractions.

### 2.8. Aquaporin-9 Quantification

**Figure 9:**
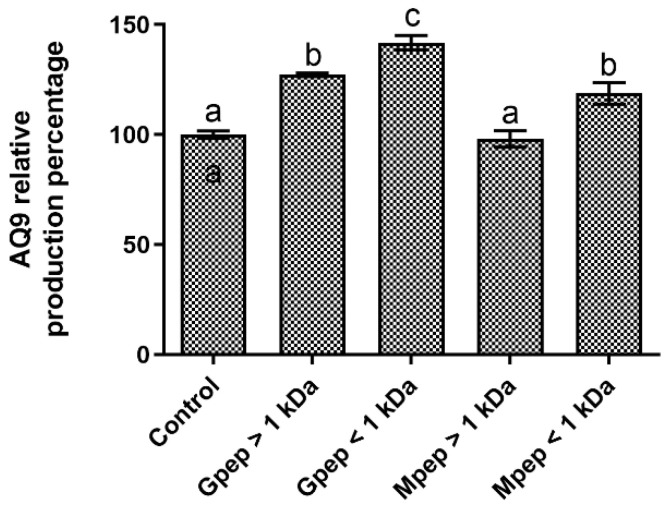
Aquaporin-9 relative percentage of production in the presence of different peptide fractions relative to the basal level (control). Different letters represent the statistically significant differences (*p* < 0.05) found between the samples tested.

The obtained data regarding the tested fractions’ capacity to modulate aquaporin-9 production in HaCat cells can be seen in Figure 9. Overall, the tested samples were, at worst, non-effective (Mpep > 1 kDa) and at best led to small, but statistically significant (*p* < 0.05) promotions of the target metabolite production. The fraction that led to highest promotions was the Gpep < 1 kDa. Additionally, it bears notice that once again, no clear pattern of response emerged—at least not one related to the studied fractions’ MW.

## 3. Discussion

Byproducts and waste valorization-based peptides applications in cosmetics is an ever-expanding area. Thus, one has to consider the different sources, with different extraction procedures and different fractions at play, which will all influence the assay outcome. Examples of peptides and peptides fractions of cosmetic interest can be found in the literature with the tyrosinase inhibiting peptides obtained from milk, wheat, or honey being examples, as well as the inhibition of collagenase by proteins obtained from hydrolyzed spirulina being other [14,15]. Presently, when one considers SYWS obtained peptide fractions, there are no records of cosmetic-related applications related to this peptide source; however, a critical analysis of the literature regarding bioactive peptides may provide some insights into the data presented here.

Antioxidant compounds have long been an integral part of cosmetic formulations as they are billed as playing important roles in human health, as well as in formulation stability. However, most traditional-use antioxidants are insoluble and hard to incorporate in formulations or instable and degrade over time [16]. One solution has been the formulation of delivery solutions capable of protecting these agents and another has been the search for alternative molecules, such as peptides, that may also possess antioxidant capacity. Bioactive peptides have long been shown to possess significant antioxidant capacity with most of the mechanisms attributed to traditional antioxidants being present in these compounds [17]. For SYWS-derived peptides, a significant antioxidant capacity has already been reported with the mechanism behind this activity not being completely understood but being believed to be linked to the fractions MW. In fact, previous works have shown that fractions below 3 kDa, which typically contain less than ten amino acid residues, presented higher antioxidant activity results than fractions with higher MWs. This behavior was also present in this work as both fractions below 1 kDa presented higher antioxidant activity than those with MWs higher than 1 kDa [18,19,20]. However, while this activity has been reported, most of the works behind it used methods such as ABTS^•+^ and FRAP and not ORAC. This limits the possibility of direct comparisons of the data obtained, as the first two are single electron transfer assays, whereas ORAC is a hydrogen atom transfer (HAT) assay; thus, two different mechanisms are at play here. Furthermore, ABTS^•+^ presents limited application for in vivo analysis as it does not register the activity of glutathione, thiols, and proteins that are HAT dominant [21]. The only works where similar settings were assayed are those of Amorim, Marques [8], which showed that peptides obtained from *S. cerevisiae* with MWs below 3 kDa had higher antioxidant activity than fractions with higher MWs, and Marson, de Castro [22], where the antioxidant activity was correlated with the peptide fraction’s hydrolysis degree and not its MW, and the activity was dependent on the enzyme–peptide bond specificity. 

Although bioactive peptide biocompatibility is a well-studied field with various examples in different cell lines, concentrations, and conditions [23], when one considers SYWS-derived bioactive peptides, the existing body of work is much more limited, no matter the target cell line assayed. Restu, Nishida [24] reported that HeLa cells retained 60% of their viability when exposed to 0.6 wt% of peptide fraction and that NIH3T3 cells retained 60% of their viability in the presence of 0.8 wt% in the same exposure conditions. Mirzaei, Mirdamadi [25] showed that peptide fractions with MWs below 3 kDa did not present deleterious effects towards Caco-2 metabolism up to 2 mg/mL and more recently, Oliveira, Pereira [7] showed that the SYWS-derived peptides used here in this work were not cytotoxic towards Caco-2 cells up to 5 mg/mL, a value well above the one obtained here. This can be easily explained through intrinsic differences between cell lines, which leads to differences in sensitivity to compounds [26,27,28,29].

When regarding the impact of SYWS peptides upon cellular metabolite production of cosmetic interest, there are no previous works that can act as reference to compare with this work, but other peptide sources have been reported with related activities. 

Traditionally, peptides for cosmetic applications have been grouped into four different categories—signal, carrier, neurotransmitter, and enzyme inhibitors—with the promotion of matrix protein targets, as the ones studied in this work, being ascribed to the first category [1,2]. When disregarding synthetic peptides and considering only those obtained from natural sources such as animal or plants, the most common ones are obtained from animals and are centered upon collagen-derived peptides, as these have been proven as being capable of promoting the production of extracellular matrix (ECM) components [30,31]. Clear examples can be found in the works of Tsai, Hsu [32], in which peptides from the proteolytic hydrolysis of collagen were capable of upregulating fibronectin and collagen type I and II in skin fibroblasts. Edgar, Hopley [33] showed that collagen-derived peptides with MWs between 0.3 and 8 kDa were capable of promoting elastin synthesis in primary dermal fibroblasts. Similarly, Kumar, Reddi [34] showed that milk-derived novel bioactive peptides enhanced extracellular collagen production in dermal fibroblasts, and the more exoteric works of Offengenden, Chakrabarti [35] and Benjakul, Karnjanapratum [36] reported that peptides with MWs under 2 kDa obtained from chicken collagen and sea bass skin, were capable of promoting type I pro-collagen and soluble collagen, respectively.

In addition to the above referenced collagen, fibronectin and elastin, the other target of cosmetic products is hyaluronic acid. For this metabolite, the most commonly taken approach is the inhibition of the hyaluronidase enzyme and not the modulation of its production. Examples of this can be seen in the literature in the works of Nakchum and Kim [37], where various squid-derived peptide fractions inhibited hyaluronidase activity and thus increased overall hyaluronic acid levels, and of Norzagaray-Valenzuela, Valdez-Ortiz [38], in which microalgae-derived peptides inhibited this enzyme activity. When one considers direct hyaluronic acid promotion, there are various works depicting the capacity of synthetic peptides to promote this metabolite production [39,40]. One of the few examples of natural peptide fractions direct activity upon hyaluronic acid can be found in the work of De Lucia, Zappelli [41], which showed that a microalgae-derived peptide fraction was capable of promoting hyaluronidase synthetase up regulation. Lastly, there is keratin and aquaporin production modulation. For keratin production, Nicolas-Espinosa, Yepes-Molina [42] showed that plant-derived bioactive peptides were capable of upregulating keratinization factors in HaCat. For aquaporin, a family of proteins involved in water transport in the skin and allow the passage of water and glycerol through the epidermis [43], De Lucia, Zappelli [41] showed that a microalgae-derived peptide fraction up-regulated the genes involved in the expression of these proteins’ family. 

Overall, the obtained results showed that the studied peptide fractions possessed potential as a possible future solution for cosmetic applications. They were not cytotoxic towards skin cell lines, and at a relatively low concentration (100 µg/mL), they were capable of modulating the various cosmetically relevant metabolites tested. Of all tested extracts the overall best performer was the Gpep > 1 kDa with consistent performance over all metabolites tested. However, this fraction possesses an advantage over the other ones tested, as the nutraceutical characterization performed by Oliveira, Pereira [7] showed that this fraction possesses a 28.2 ± 7.9% sugar content, a family of compounds known to promote cellular metabolism [44]. These results, while preliminary, open the potential for a future valorisation stream for SYWS, thus adding value to a byproduct usually applied in animal feeding or still discarded. 

## 4. Materials and Methods

### 4.1. Peptide Fraction Production

Peptide fraction production and characterization has been described in a previous work by Oliveira, Pereira [7]. Peptide rich extracts were obtained from engineered spent yeast (*Saccharomyces cerevisiae*) used in Amyris facilities to produce β-farnesene (Emeryville, CA, USA). Waste streams of β-glucan (Gpep) and mannan (Mpep) extractions from spent yeast underwent 1 kDa cut-off membrane ultrafiltration using a Amicon^®^ stirred cell model (Merck KGaA, Darmstadt, Germany) in order to obtain fractions with different MWs. At the end of the process, samples were freeze-dried (Freeze-dryer Alpha 2–4 LSCbasic, Martin Christ, Osterode am Harz, Germany) and stored for later use.

### 4.2. Antioxidant Activity

Peptide fraction antioxidant activity was assayed through oxygen radical absorbance capacity (ORAC) assay as previously described [45]. Briefly, in black 96-well microplates, peptide fractions at the desired concentrations and 75 mM phosphate buffer (pH 7.4; blank) or Trolox (10 to 80 µmol/L, standard) were added to fluorescein (70 nM). The plate was then pre-incubated for 10 min at 37 °C during 10 min, following which time AAPH solution (48 mM) was added to the wells and fluorescence (Ex: 485 nm; Em: 528 nm) was read every minute for 120 min using a microplate reader (Synergy H1, Biotek, Santa Clara, CA, USA). Results were obtained through plotting the data against a calibration curve, and they were expressed as micromoles of Trolox equivalent per gram of extract. All assays were performed in triplicate. 

### 4.3. Cell lines and Culture Conditions

Two different cell lines were assayed throughout this work. Human keratinocytes (HaCat (CLS 300493, Eppelheim, Germany)) and primary dermal fibroblasts, normal, human, adult (HDFa (ATCC PCS-201-012, VA, USA)). HaCat cells were cultured at 37 °C in a humidified atmosphere of 95% air and 5% CO_2_ as monolayers using Dulbecco’s modified Eagle’s medium (DMEM) with 4.5 g/L glucose, L-glutamine without pyruvate (ThermoScientific, MA, USA) containing 10% fetal bovine serum (ThermoScientific, MA, USA) and 1% (*v/v*) Penicillin-Streptomycin-Fungizone (ThermoScientific, MA, USA). HDFa cells were cultured at 37 °C in a humidified atmosphere of 95% air and 5% CO_2_, as monolayers, using fibroblast growth medium (Sigma-Aldrich, St. Louis, MO, USA). Keratinocytes were used between passages 33 and 44 and fibroblasts were used between passages 6 and 9.

### 4.4. Evaluation of the Peptide’s Fractions Cytotoxicity

Cytotoxicity assays were performed accordingly to the ISO 10993-5:2009 standard as previously described by Costa, Pereira [29]. Briefly, cells were grown to 80–90% confluence, detached using TrypLE Express, and seeded at 1 × 10^4^ cells/well in a 96-well microplate. After 24 h, the media was carefully removed and replaced with culture media supplemented with test samples at the selected concentrations. Dimethyl sulfoxide at 10% (*v/v*) in culture media was used as a death control and plain culture media was used as growth control. After 24 h of incubation, Presto Blue was added to each well and the plate was re-incubated. Following this, fluorescence (Ex: 560 nm; Em: 590 nm) was measured using a microplate reader. All assays were performed in quadruplicate. Results were given in terms of percentage of cell metabolism inhibition.

### 4.5. Pro-Collagen I α I Biosynthesis and Quantification

Pro-collagen I α I content in HDFa cells was determined as previously described by Costa, Pereira [29]. Briefly, cells were seeded at 5 × 10^5^ cells/well in a 6-well microplate and incubated for 24 h at 37 °C in a humidified atmosphere of 95% air and 5% CO_2_. After 24 h, culture media was carefully replaced with peptides samples (100 µg/mL) supplemented media, and the plate was re-incubated for another 24 h. All samples were assayed in triplicate. At the end of the assay, supernatants were collected, centrifuged to remove debris, and stored for further analysis. Cells were washed with chilled PBS and cell extracts were obtained using the Human Pro-Collagen I alpha1 Catchpoint SimpleStep Elisa (Abcam, Cambridge, MA, USA) according to the manufacturer’s instructions. The protein contents of aliquots of cell extracts were determined using the BCA Pierce Assay Kit (ThermoScientific, MA, USA). All samples were analyzed in sextuplicate.

Pro-collagen I α I contents in supernatants and cell extracts were determined by enzyme-linked immunosorbent assay (ELISA) using the Abcam kit described above in accordance with the manufacturer’s instructions. Pro-collagen I α I values in cell extracts were obtained in pg of pro-collagen1 alpha1/ng protein, whereas in-cell supernatants were obtained in pg of pro-collagen1 alpha1/mL sample. To diminish the variability associated with any kind of proteomic-based assay, results were expressed in relative percentage of production relative to the pro-collagen I α I levels in the basal (non-stimulated) control. Pro-collagen content of the basal control was set to 100%. All samples were assayed in quadruplicate.

### 4.6. Hyaluronic acid Biosynthesis and Quantification

HaCat and HDFa cells were seeded at 5 × 10^5^ cells/well in a 6-well microplate and incubated for 24 h at 37 °C in a humidified atmosphere of 95% air and 5% CO_2_. After 24 h, the culture media was carefully replaced with peptide fractions (100 µg/mL) supplemented media and the plate re-incubated for another 24 h. All samples were assayed in triplicate. At the end of the assay, supernatants were collected, centrifuged to remove debris and stored for further analysis. 

Hyaluronic acid content in supernatants was determined by enzyme-linked immunosorbent assay (ELISA) using the hyaluronic acid Elisa Kit (BioVision E4626-100) in accordance with the manufacturer’s instructions. Hyaluronic acid values in cell supernatants were obtained in ng of hyaluronic acid/mL of sample. To diminish the variability associated with any kind of proteomic-based assay results were expressed in relative percentage of production relative to the hyaluronic acid levels in the basal (non-stimulated) control. The hyaluronic acid content of the basal control was set to 100%. All samples were assayed in quadruplicate.

### 4.7. Fibronectin Biosynthesis and Quantification

HDFa cells were seeded at 5 × 10^5^ cells/well in a 6-well microplate and incubated for 24 h at 37 °C in a humidified atmosphere of 95% air and 5% CO_2_. After 24 h, the culture media was carefully replaced with peptide fraction (100 µg/mL) supplemented media, and the plate was re-incubated for another 24 h. All samples were assayed in triplicate. At the end of the assay, supernatants were collected, centrifuged to remove debris, and stored for further analysis. Fibronectin content in the supernatants was determined by enzyme-linked immunosorbent assay (ELISA) using the Human Fibronectin Catchpoint SimpleStep Elisa kit (Abcam ab229398) in accordance with the manufacturer’s instructions. Fibronectin values in cell supernatants were obtained in pg of fibronectin/mL of sample. To diminish the variability associated with any kind of proteomic-based assay, results were expressed in relative percentage of production relative to the fibronectin levels in the basal (non-stimulated) control. The fibronectin content of the basal control was set to 100%. All samples were assayed in quadruplicate.

### 4.8. Cytokeratin-14 Biosynthesis and Quantification

HaCat cells were seeded at 5 × 10^5^ cells/well in a 6-well microplate and incubated for 24 h at 37 °C in a humidified atmosphere of 95% air and 5% CO_2_. After 24 h, the culture media was carefully replaced with peptide fraction (100 µg/mL) supplemented media and the plate was re-incubated for another 24 h. At the end of the assay, the supernatants were collected, centrifuged to remove debris, and stored for further analysis. All samples were assayed in triplicate.

Cytokeratin 14 content in supernatants was determined by enzyme-linked immunosorbent assay (ELISA) using the Human Cytokeratin 14 SimpleStep ELISA kit (Abcam ab226895) in accordance with the manufacturer’s instructions. Cytokeratin 14 values in cell supernatants were obtained in pg of cytokeratin 14/mL of sample. To diminish the variability associated with any kind of proteomic-based assay, results were expressed in relative percentage of production relative to the cytokeratin 14 levels in the basal (non-stimulated) control. Cytokeratin-14 content of the basal control was set to 100%. All samples were assayed in quadruplicate.

### 4.9. Elastin Biosynthesis and Quantification

HDFa cells were seeded at 2.5 × 10^5^ cells/well in a 6-well microplate and incubated for 24 h at 37 °C in a humidified atmosphere of 95% air and 5% CO_2_. After 24 h, the culture media was carefully replaced with peptide fraction (100 µg/mL) supplemented media, and the plate was re-incubated for another 24 h. At the end of the assay, cells were detached into suspension, centrifuged, and the cell pellet was kept for further analysis. All samples were assayed in triplicate.

Elastin content in cell pellets was determined using the Fastin Elastin (Biocolor, Co antrim, UK) kit in accordance with the manufacturer’s instructions. Elastin values in cell pellets were obtained in ng of elastin/mL of sample. To diminish the variability associated with any kind of proteomic-based assay, results were expressed in relative percentage of production relative to the elastin levels in the basal (non-stimulated) control. Elastin content of the basal control was set to 100%. All samples were assayed in quadruplicate.

### 4.10. Aquaporin 9 Biosynthesis and Quantification

HaCat cells were seeded at 2.5 × 10^5^ cells/well in a 6-well microplate and incubated for 24 h at 37 °C in a humidified atmosphere of 95% air and 5% CO_2_. After 24 h, the culture media was carefully replaced with peptide fraction (100 µg/mL) supplemented media, and the plate was re-incubated for another 24 h. At the end of the assay, cells were detached into suspension, centrifuged, and the supernatant was kept for further analysis. All samples were assayed in triplicate.

Aquaporin 9 content in supernatants was determined by enzyme-linked immunosorbent assay (ELISA) using the Human Aquaporin 9 ELISA kit (MyBioSource; San Diego, USA) in accordance with the manufacturer’s instructions. Aquaporin 9 values in cell pellets were obtained in pg of AQ9/ng of protein. To diminish the variability associated with any kind of proteomic-based assay, results were expressed in relative percentage of production relative to the aquaporin 9 levels in the basal (non-stimulated) control. Elastin content of the basal control was set to 100%. All samples were assayed in quadruplicate.

### 4.11. Statistical Analysis

One-phase decay, linear, and four-parameter regressions for ELISA analysis were performed using GraphPad Prism 6 software (San Diego, CA, USA). Statistical analysis was performed using IBM SPSS Statistics v21.0.0 (New York, NY, USA) software. As the data followed a normal distribution, One-way ANOVA coupled with Tukey’s post hoc test was used to assess the differences between the results observed with differences considered significant for *p*-values below 0.05.

## Data Availability

The data presented in this study are available on request from the corresponding author. The data are not publicly available due to confidentiality agreements.
